# Genome Sequence of Vibrio cholerae Strain RFB16, Isolated from North Park Lake in Allegheny County, Pennsylvania

**DOI:** 10.1128/MRA.00111-20

**Published:** 2020-03-05

**Authors:** Rachel F. Bina, James E. Bina, Yuding Weng

**Affiliations:** a University of Pittsburgh School of Medicine, Department of Microbiology and Molecular Genetics, Pittsburgh, Pennsylvania, USA; University of Rochester School of Medicine and Dentistry

## Abstract

Vibrio cholerae is an aquatic organism and facultative human pathogen that typically resides in coastal areas and brackish water. Here, we report the complete genome sequence of V. cholerae strain RFB16, which was isolated from a freshwater lake in southwestern Pennsylvania.

## ANNOUNCEMENT

Vibrio cholerae is an aquatic organism and facultative human pathogen that causes the pandemic disease cholera (1). Cholera is often associated with poor sanitation, which contributed to a cholera epidemic in Pittsburgh, Pennsylvania, in the 1800s (2). In this study, we report the genome sequence of a V. cholerae strain that was isolated from a freshwater lake near Pittsburgh, Pennsylvania. The genome of this strain will be important for studying the evolution of V. cholerae strains in the environment.

*Vibrio* species were isolated from North Park Lake in Allegheny County, Pennsylvania (40°35′52.4ʺN, 79°59′52.5ʺW), by collecting 1-liter surface water samples. *Vibrio* ssp. in the water samples were enriched by passing the water through a sterile 0.22-μm filter and incubating the filters in 50 ml of alkaline peptone water (APW) overnight at room temperature (3). The next day, 50 μl of the APW was transferred to 10 ml of fresh APW, and the cultures were incubated at 30°C for 4 hours before aliquots were spread onto the surface of thiosulfate-citrate-bile salts-sucrose (TCBS) agar plates and incubated overnight at 30°C. The following day, large yellow colonies (∼3 mm) that were consistent with V. cholerae were purified by streaking onto TCBS agar. The resulting colonies were examined for vibrio-shaped cells by phase-contrast microscopy, and one representative colony, named RFB16, was retained and used for subsequent analyses.

RFB16’s species determination was confirmed by PCR for the V. cholerae species-specific diagnostic marker *ompW* gene (4). The RFB16 genome was sequenced by SNPsaurus (Eugene, OR) using whole-genome PacBio sequencing. Briefly, genomic DNA was isolated using the MasterPure kit (Epicentre) according to the manufacturer’s instructions. The genomic DNA was then processed according to the PacBio “preparing multiplexed microbial SMRTbell libraries for the PacBio Sequel system” protocol and sequenced with SMRT Link 6.0 sequencing chemistry and a library size selected for fragments of ≥7 kb with a 30-hour read time using a fraction of a single-molecule real-time (SMRT) cell. This resulted in 193,463 sequencing reads with a 13,925-bp mean read length, which generated 2.2 × 109 total bases that were *de novo* assembled and polished using Flye 2.4.1 (5) with default settings. This generated three closed circular contigs of 2,948,589 bp, 1,140,710 bp, and 49,113 bp, respectively. The assembly quality was assessed using BUSCO 3 and was 98.0% complete, with 98.0% of the genome single copy and 0.0% duplicated (6).

The 2.95- and 1.14-Mb contigs represented the large and small chromosomes (7) and had a GC content of 47.5% each. The 49-kb contig represented a plasmid with a GC content of 46.9%. The genome was annotated using the NCBI Prokaryotic Genome Annotation Pipeline (PGAP), which identified genes encoding the virulence regulator ToxR and outer membrane proteins OmpU and OmpW but not genes encoding cholera toxin or the toxin coregulated pilus (8).

### Data availability.

The RFB16 genome sequence was deposited in GenBank under BioProject number PRJNA560225 with the accession numbers CP043554, CP043556, and CP043555. The raw sequencing results were deposited in the NCBI SRA database under number PRJNA560225.
